# Supplementary material for: A Mouse Model of Mild Clostridioides difficile Infection for the Characterization of Natural Immune Responses
Source: Microorganisms. 2024 Sep 24;12(10):1933. doi: 10.3390/microorganisms12101933 (PMC11509167; doi:10.3390/microorganisms12101933)
Supplement: Supplementary file 1 [file microorganisms-12-01933-s001.zip › ARRIVE_Checklist_Filled.pdf]

Checklist ARRIVE

1. Study Design

This study describes and validates a mouse model of primary mild *Clostridioides difficile* infection (CDI). The focus of the study is to evaluate the kinetics of humoral (IgG, IgM) and mucosal (IgA) immune responses against toxins (TcdA/TcdB) and surface proteins (SlpA/FliC). The study involves gut microbiota analysis to assess dysbiosis caused by antibiotics.

2. Sample Size

A total of 78 C57BL/6 female mice were used, grouped for endpoint times (D-6, D2, D7, D14, D21, D28, and D56) to evaluate immune response and microbiota changes. Each group was randomly assigned based on these time points.

3. Inclusion and Exclusion Criteria

Inclusion criteria: Female C57BL/6 mice, 6 weeks old, with no prior colonization by *C. difficile*, obtained from pathogen-free environments. Exclusion criteria: Mice with pre-existing *C. difficile* colonization or those displaying poor health at baseline.

4. Randomization

Mice were randomly assigned to experimental groups based on the different time points (days of sample collection).

5. Blinding

Outcome assessors (those measuring immune responses) were blinded to the group allocations. However, the investigators responsible for the animal management and sample collection were not blinded.

6. Outcome Measures

Primary outcomes included weight loss, mortality, duration of *C. difficile* colonization, and immune responses (IgG, IgM, and IgA) specific to toxins and surface proteins. Gut microbiota composition changes were also evaluated.

7. Statistical Methods

Statistical analyses included descriptive statistics, Kruskal-Wallis tests followed by Wilcoxon tests for non-parametric comparisons. Pairwise PERMANOVA was used for beta diversity analyses, with 999 permutations. Correction for multiple comparisons was applied using the Bonferroni method.

8. Experimental Procedures

Dysbiosis was induced using an antibiotic cocktail administered in drinking water, followed by a single clindamycin injection. Mice were challenged with 10^5 CFU of *C. difficile*

(vegetative forms) via oral gavage. Samples of feces, blood, and cecal contents were collected at various time points for analysis.

9. Animal Housing and Husbandry

Mice were housed in groups of 4-5 per cage, in pathogen-free facilities with a controlled environment. Cages, bedding, water, and food were autoclaved. Mice had ad libitum access to water and food.

10. Ethical Statement

All animal procedures were approved by the University of Paris-Sud Ethics Committee (APAFIS#23414-2019121910116284). The study adhered to national and European guidelines for animal care and use.
