# Supplementary figures and images for: A Mouse Model of Mild Clostridioides difficile Infection for the Characterization of Natural Immune Responses
Source: Microorganisms. 2024 Sep 24;12(10):1933. doi: 10.3390/microorganisms12101933 (PMC11509167; doi:10.3390/microorganisms12101933)

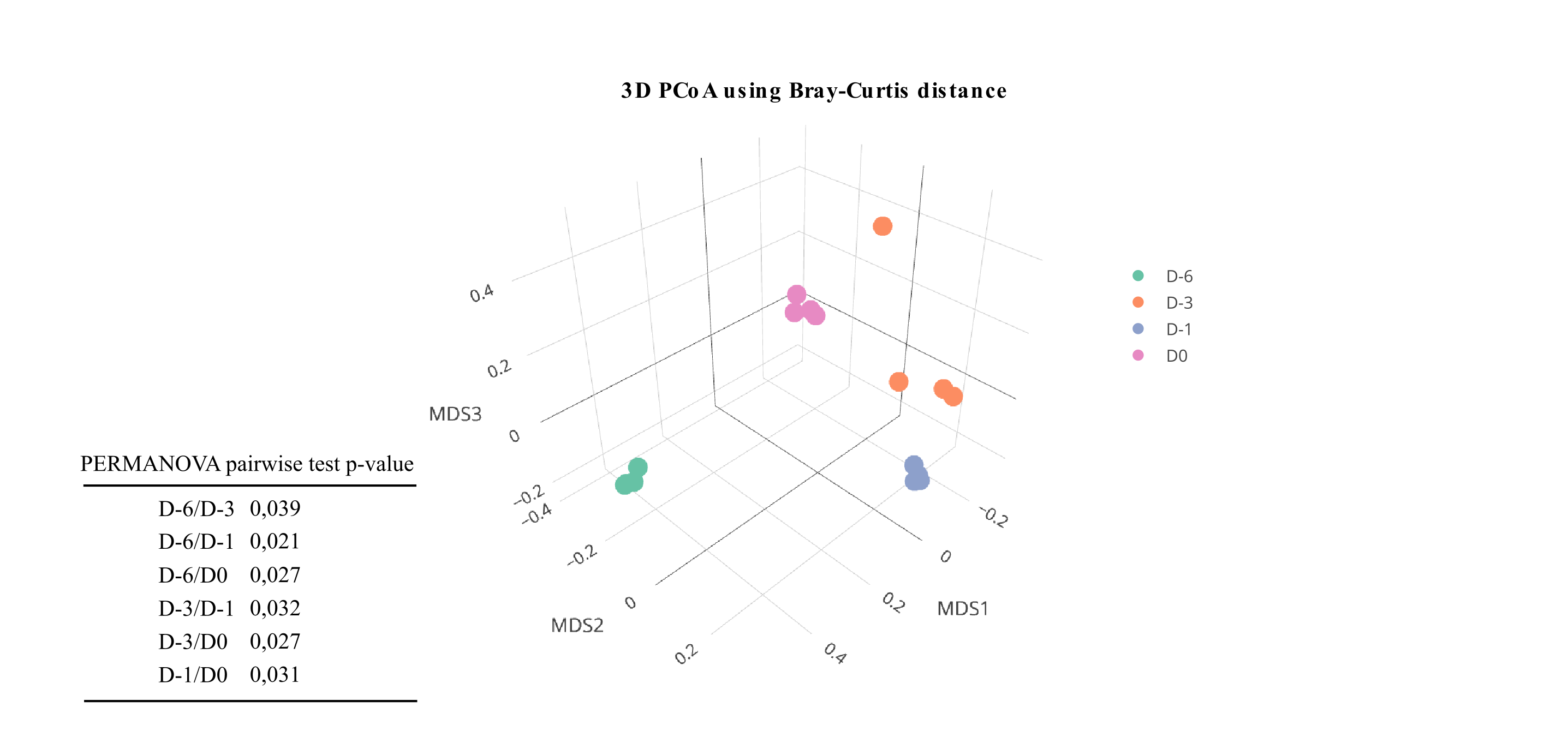

Supplement: Supplementary file 1 [file microorganisms-12-01933-s001.zip › Figure S1_PCoA3D.tiff]

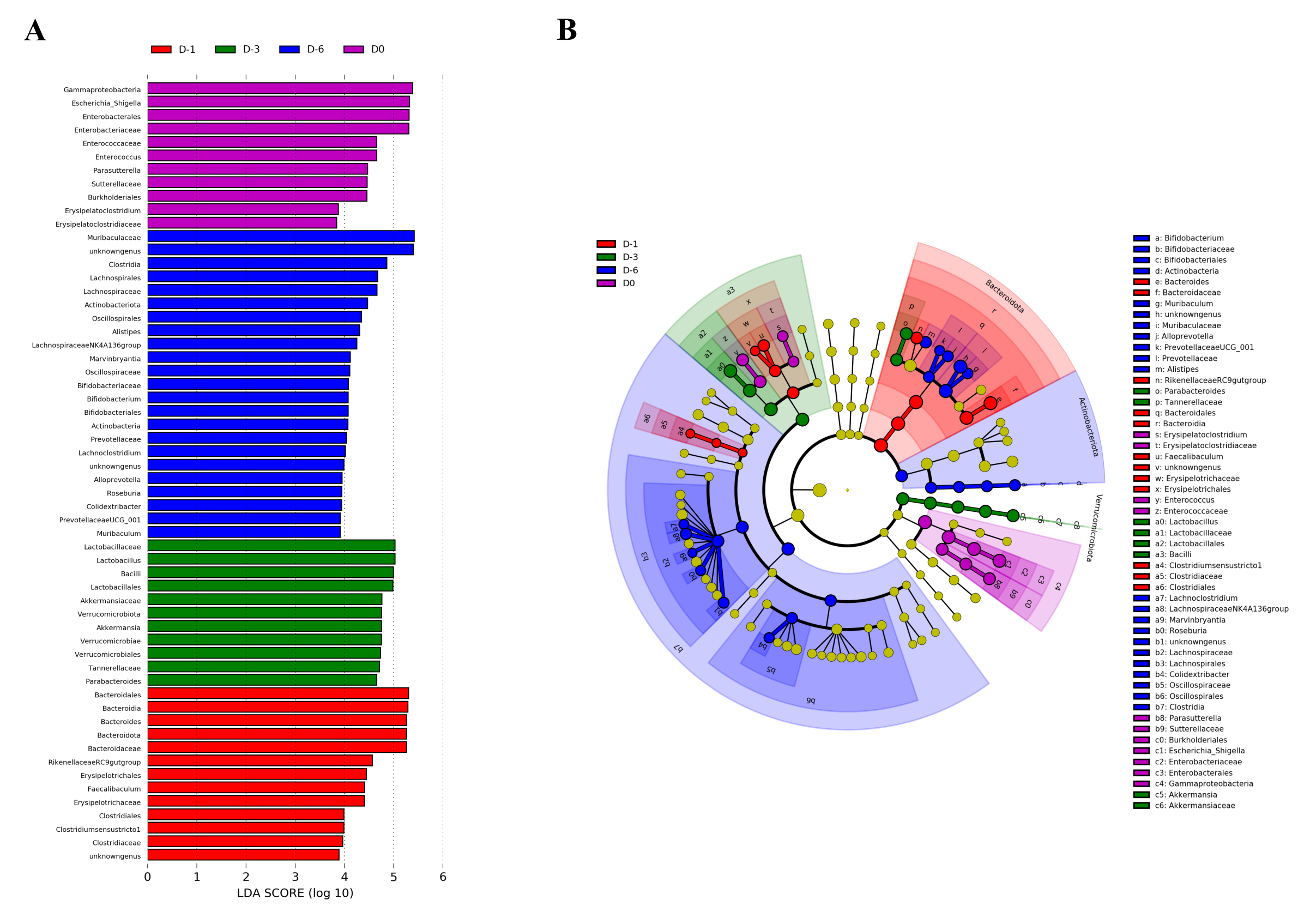

Supplement: Supplementary file 1 [file microorganisms-12-01933-s001.zip › Figure S2_LDA-cladogram.tiff]
